# Genetic basis of offspring number–body weight tradeoff in *Drosophila melanogaster*

**DOI:** 10.1093/g3journal/jkab129

**Published:** 2021-04-20

**Authors:** Jamilla Akhund-Zade, Shraddha Lall, Erika Gajda, Denise Yoon, Julien F Ayroles, Benjamin L de Bivort

**Affiliations:** 1 Department of Organismic and Evolutionary Biology, Center for Brain Science, Harvard University, Cambridge, MA 02138, USA; 2 Department of Ecology and Evolutionary Biology, Lewis-Sigler Institute for Integrative Genomics, Princeton University, Princeton, NJ 08544, USA

**Keywords:** Drosophila melanogaster, offspring number, body weight, fecundity, GWAS, DGRP, life history

## Abstract

*Drosophila melanogaster* egg production, a proxy for fecundity, is an extensively studied life-history trait with a strong genetic basis. As eggs develop into larvae and adults, space and resource constraints can put pressure on the developing offspring, leading to a decrease in viability, body size, and lifespan. Our goal was to map the genetic basis of offspring number and weight under the restriction of a standard laboratory vial. We screened 143 lines from the *Drosophila* Genetic Reference Panel for offspring numbers and weights to create an “offspring index” that captured the number *vs* weight tradeoff. We found 18 genes containing 30 variants associated with variation in the offspring index. Validation of *hid*, *Sox21b*, *CG8312*, and *mub* candidate genes using gene disruption mutants demonstrated a role in adult stage viability, while mutations in *Ih* and *Rbp* increased offspring number and increased weight, respectively. The polygenic basis of offspring number and weight, with many variants of small effect, as well as the involvement of genes with varied functional roles, support the notion of Fisher’s “infinitesimal model” for this life-history trait.

## Introduction

Aspects of life history, such as fecundity, lifespan, and body size, can affect an organism’s evolutionary fitness. In *Drosophila melanogaster*, the genetics, plasticity, and evolution of life-history traits have been extensively studied (Flatt 2020). *Drosophila* fecundity, measured through egg-laying behavior, was previously shown to have a strong genetic component that differs between young and old flies (Rose and Charlesworth 1981; Tatar *et al.* 1996; Leips *et al.* 2006; Durham *et al.* 2014) and is also influenced by temperature and nutrition (Chippindale *et al.* 1993; Nunney and Cheung 1997). Fecundity interacts with a number of physiological processes. In support of an energy allocation model of life history (van Noordwijk and de Jong 1986), fecundity has been shown to tradeoff with longevity (Tatar *et al.* 1996; Djawdan *et al.* 1996), indicating that investment into the next generation can come at a cost to somatic maintenance via a transfer of energy reserves. A genome-wide association study (GWAS) revealed that age-specific fecundity is associated with variants present across a large set of candidate genes, enriched for genes involved in development, morphogenesis, neural function, and cell signaling (Durham *et al.* 2014). Connecting fecundity with neural function, quantitative trait locus and deficiency mapping revealed that expression of a *Drip* aquaporin in corazonin neurons was positively correlated with fecundity by modulating the neurohormone balance between corazonin and dopamine (Bergland *et al.* 2012).

While the vast majority of *Drosophila* fecundity studies have used egg production as a measure of fecundity, the number of eggs laid may not translate perfectly to viable offspring due to potential mortality at the multiple developmental stages. Under both natural and laboratory conditions, larvae must contend with a finite space and resource limitations given the constraints of the rotting food substrate (Grimaldi and Jaenike 1984; Prout and Barker 1989) or culture media, as well as competition between larvae. Increased larval density decreases egg-to-adult viability (Barker 1973; Scheiring *et al.* 1984; Horváth and Kalinka 2016), body size (Miller and Thomas 1958; Scheiring *et al.* 1984; Economos and Lints 1984; Santos *et al.* 1994; Baldal *et al.* 2005), and longevity (Moghadam *et al.* 2015), while increasing development time (Scheiring *et al.* 1984; Economos and Lints 1984; Santos *et al.* 1994; Horváth and Kalinka 2016) and lowering starvation resistance (Baldal *et al.* 2005). Highly fecund flies that lay a large number of eggs may end up negatively affecting their offspring due to the increased larval density. On the other end of the spectrum, flies producing fewer eggs may have large offspring capable of weathering stress (Djawdan *et al.* 1998; Jumbo-Lucioni *et al.* 2010) that go on to produce more of their own offspring. But the fitness associated with these strategies is context-dependent. For example, a strategy of fewer, larger offspring may be effective amidst a high density of competitors, but ineffective in the presence of high predation. Moreover, a critical density of larvae is needed to engage in cooperative food-burrowing (Dombrovski *et al.* 2017), with cooperative food-burrowing more likely to occur in conspecifics of high relatedness (Khodaei and Long 2019). Taken together, these pressures mean that (1) finite space and resource limitation and (2) context-dependent changes in associated fitness likely impose a tradeoff between the number of offspring and their body phenotypes.

Given that these tradeoffs come into play before and after egg-laying, we set out to determine if there is a genetic basis for a genotype’s position on the offspring number-quality tradeoff, by measuring progeny phenotypes under standardized resource conditions. We used the standard laboratory food vial to impose both a space and food limitation on the developing offspring. As a measure of quality, we measured the wet weight of recently eclosed offspring; increased body weight is correlated with increased starvation resistance (Jumbo-Lucioni *et al.* 2010), increased nutrient stores, and increased immunity (Unckless et al. 2015), which indicate an investment in somatic maintenance. We scored lines from the *Drosophila* Genetic Reference Panel or DGRP (Mackay *et al.* 2012) for numbers of adult offspring and their weight. In a GWAS, we found candidate genes with variants significantly associated with a combined metric of offspring number and weight. Mutation of these genes, in most cases, caused lethality or impaired survival at the adult stage, but in other cases, shifted the balance between offspring weight and number. Beyond these top hit genes, we found evidence that many genes may contribute small effects to this tradeoff phenotype.

## Materials and methods

### Drosophila stocks and husbandry

We analyzed 143 lines from the DGRP (Mackay *et al.* 2012). All stocks were maintained in incubators at 23°C, 12L:12D cycle and reared on a yeast, cornmeal, and dextrose media (23 g yeast/L, 30 g cornmeal/L, 110 g dextrose/L, 6.4 g agar/L, and 0.12% Tegosept). Experiments were carried out in polystyrene narrow culture vials (25 × 95 mm, #32-109, Genesee Scientific). Mutant lines for validation were obtained from the Exelixis collection at Harvard Medical School (Boston, MA, USA) (Thibault *et al.* 2004). The *w^1118^* (#6326) genetic background was obtained from the Bloomington Drosophila Stock Center (Bloomington, IN, USA) as a control for the candidate gene validation mutant lines, as it is the progenitor line for the Exelixis gene disruption collection.

### Phenotypic measurements of DGRP lines

Bottles were seeded with 15 females and 15 males in 12L:12D to generate the parent flies that would go on to lay eggs for the experiment. Ten females and five males (2–5 days old) from the parent flies were placed in each of the three vials (along with ∼30 grains of dry yeast) and left to oviposit for 2 days at 23°C, 12L:12D. The parent flies were removed, and the vials were kept at 23°C, 12L:12D until progeny began to eclose. From the start of eclosion, all of the vials were examined every day over the course of 10 days. The number of females and males for each vial was recorded, as well as the total wet weight (to 0.1 mg accuracy) of the females and the total combined wet weight of the females and males. Male weight was calculated by subtracting the female weight from the total. Ten days were chosen to measure as many offspring as possible without measuring any flies from the subsequent generation. One hundred and forty-three lines were tested in two batches. One hundred and thirty-four lines were tested in the first batch. The second batch included the 35 lines that did not have all three vial replicates completed in the first batch, and 9 lines that were not tested at all in the first batch.

### Calculating genetic correlation among traits

Genetic correlation and its 95% highest posterior density interval (HPDI) among the four measured phenotypes were calculated using a multivariate normal mixed model with the brms package in R (v2.8.0). The four phenotypes were the response variables and the random effect (correlated across phenotypes) of DGRP line was the predictor. Mass phenotypes were log-transformed prior to modeling.

### Genome-wide association mapping for offspring index

The four phenotypes measured in this screen were the total number of females (males) eclosed and the average weight of a female (male) fly. Since lines were assayed in two batches, the 35 lines that were tested in both batches were used to check for a batch effect. The batch effect for each of the four phenotypes (number of ♀ = −17, number of ♂ = −22, mean ♀ weight = 0.13, mean ♂ weight = 0.12) was corrected by applying an offset (difference of mean phenotype between the first and second batch) calculated from the overlapping set of lines.

For offspring total counts, a random intercept linear model was used to calculate the random effect of each DGRP line on vial phenotype (each line had three vial replicates):

$$Y_{a}=\mu +\mathrm{lin}e_{a}\;+\varepsilon ,$$

where $Y_{a}$ is the vial phenotype measure (total offspring number or mean weight) for line *a*, $\mathrm{lin}e_{a}$ is the random effect of line *a*, and ε is the error term. For mean vial offspring weights, a random intercept generalized linear model was used (model formula as above), assuming a gamma distribution of mean weights and a logarithm link function. The LME4 package (v1.1-21) in R (v3.5.3) was used for modeling. To estimate broad sense heritability of the vial phenotype, we used the R package brms (v2.8.0) and our models to estimate the fraction of variance explained by line out of all sources of variance (among-line and among-vial variance), as well as the uncertainty in the estimate from the 95% HPDI.

Since we were interested in a single metric to summarize the number of offspring and their average weight, we used *prcomp* with scaling in R’s factoextra package (v1.0.5) to generate the principal components of the dataset. The first principal component explained 71% of the variance of the dataset, so we chose to use the value of the rotated data (line phenotype values multiplied by the rotation values/loadings of the first principal component) as our summary phenotype, which we called the offspring index (Supplementary Table S3).

We used the DGRP2 webtool (Huang *et al.* 2014) to perform a mapping of variants associated with the offspring index and each of the four phenotypes. The webtool controls for inversions and *Wolbachia* infection status prior to mapping. We chose a significance threshold of *P* < 1E−5 to identify variants for further consideration. A *χ*^2^ test was run to compare the observed chromosomal distribution of variants to the expected distribution given the proportion of all segregating variants on each chromosome in the DGRP dataset (Huang *et al.* 2014). λ_median_ was calculated as the median of the *χ*^2^ test statistics for 1,896,156 DGRP variants divided by the expected median of the *χ*^2^ distribution.

### Variant effect size estimation

Variant effect sizes were estimated for 1,907,562 DGRP variants on each of the four measured traits using a linear regression of measured phenotype against DGRP genotypes at a particular variant (0 = homozygous reference, 1 = heterozygous, 2 = homozygous alternate).

$$Y=\mu +var_{\mathrm{alt}}+\varepsilon ,$$

where Y is the *Wolbachia* and inversion adjusted phenotype measure, $var_{\mathrm{alt}}$ is the fixed effect of the alternate allele, and ε is the error term. The sign of the final variant effect size was flipped if the reference allele was the major allele in the DGRP mapping population to match the webtool effect size calculation of one-half of the difference between means of the major and minor alleles (Huang *et al.* 2014). The model was implemented in Python 3.8 with scikit-learn v0.24.1.

### Parental density analysis

Six DGRP lines were chosen from the overall screen based on their offspring index values: two lines with an extreme negative index (RAL 176: −3.2, RAL 327: −2.9), two lines with an index around zero (RAL 49: −0.04, RAL 350: −0.22), and two lines with a highly positive index (RAL 812: +3.6, RAL 894: +3.8). Five density conditions were used: 1♀, 1♂; 5♀, 1♂; 10♀; 5♂, 25♀; 10♂; and 50♀, 20♂. Lines were reared in bottles at 23°C, in incubators on a 12L:12D light cycle, and a minimum of 5 (maximum of 10) replicates per line-density combination were set up, with the exception of RAL 894 at the highest density, where no replicates were set up due to an insufficient number of flies. Parent flies were 3–5 days old at the time of experiment set-up and the egg-laying conditions were the same as the DGRP phenotype screen. Given the delayed eclosion of offspring in the high-density treatments, an extended window of 25 days was used to evaluate offspring phenotypes. Daily records after the 12th day were monitored for increases in offspring number that could be indicative of a large number of offspring from the next generation, but as flies were removed daily from the vials, likely even before mating, a significant influence of next-generation offspring on the counts was deemed unlikely.

To estimate the impact of DGRP line and density on offspring counts, we used a generalized linear model, assuming a negative binomial distribution of the response along with a logarithm link function. A negative binomial model was used because of the large spread and right-skew of the offspring count distribution that made a regular linear model a poor fit. For offspring weight, a generalized linear model with a gamma response distribution and logarithm link function was used. The formula for both models was as follows:

$$Y_{ab}=\mu +\mathrm{lin}e_{a}+\mathrm{densit}y_{b}\;+\mathrm{lin}e_{a}*\mathrm{densit}y_{b}+\varepsilon ,$$

where $Y_{ab}$is the phenotype measure for a particular line-density combination, $\mathrm{lin}e_{a}$ is the fixed effect of line *a*, $\mathrm{densit}y_{b}$ is the fixed effect of a density treatment *b*, $\mathrm{lin}e_{a}*\mathrm{densit}y_{b}$ is the interaction term, and ε is the error term. DGRP line and density were treated as ordered factors in the model. DGRP lines were treated as ordered factors since the lines for this experiment were chosen to span the range of offspring index values. The lines were coded from 1 to 6: line 1 with the most negative offspring index and line 6 with the most positive offspring index. As above, the LME4 package was used, in addition to the MASS package (v7.3-51.1) for the negative binomial model. To evaluate the significance of a predictor, a likelihood ratio *χ*^2^ test using the *anova* function in R’s STATS (v3.5.3) package was used to compare models with and without the predictor.

### Validation of candidate genes

Mutant lines for validation of candidate genes were obtained from the Exelixis collection^24^ for six genes containing variants associated with the offspring index at a *P *<* *1E−5 threshold. The mutant lines were as follows: PBac{PB}*hid^c01591^*, PBac{WH}*Sox21b^f06429^*, PBac{WH}*Rbp^f07217^*, PBac{RB}*CG8312^e01204^*, PBac{WH}*CG8312^f02825^*, PBac{WH}*mub^f02647^*, PBac{WH}*Ih^f01485^*, and PBac{RB}*Ih^e01599^* (#17970 BDSC). All lines were made homozygous for the insertion prior to testing. The genetic background for this gene disruption panel was *w^1118^*. To generate the parental flies for each mutant line and the control, ∼30 females and 10 males were placed in bottles at 22°C, 12L:12D to lay for 7–10 days to generate the experimental flies. Ten females and five males from the experimental flies were put into a single vial with ∼30 yeast grains (10 replicates per mutant line) and allowed to lay for 2 days at 22°C, 12L:12D. The parental flies were removed, and the progeny were phenotyped the same way as for the DGRP phenotype screen. The validations were done in two batches staggered by 1 week. There was no significant batch effect, so replicates were combined across batches. Mutant lines were compared to the *w^1118^* genetic background control using Dunnett’s test with a family-wise confidence level of 95%. Offspring index for the mutant lines was calculated using the principal component loadings calculated from the DGRP data.

### Data availability

Data and analysis code are available at https://zenodo.org/record/4671125 and http://lab.debivort.org/genetic-basis-of-offspring-number/. Offspring index GWAS QQ plots and LD matrix are reported in Supplementary Figure S1; heritability estimates for measured phenotypes in Supplementary Table S1; GWAS results for all four measured phenotypes in Supplementary Table S2; Principal Component Analysis (PCA) variance proportion explained and loadings in Supplementary Table S3; correlation in gene expression among top hits and between gene expression and phenotype in Supplementary File S1. Supplementary material is available at figshare: https://doi.org/10.25387/g3.14403323.

## Results

### Genome-wide association mapping for offspring life-history index

**Figure 1:**
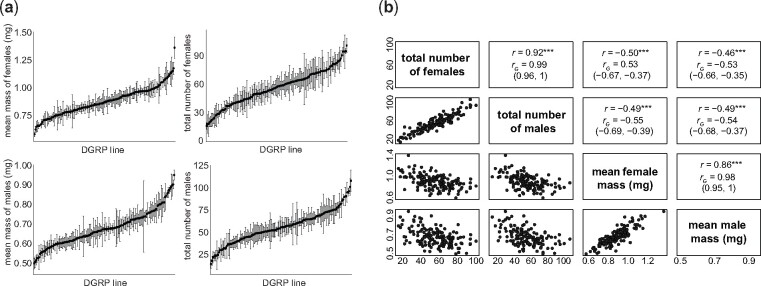
DGRP lines show variation in offspring number and weight. (A) Plot of phenotypes measured (± 1 SE) with the DGRP lines sorted by the mean value for each phenotype (three replicates/line). (B) Correlation matrix of the phenotypes measured. Points are DGRP genotypes; *r* = phenotypic Pearson correlation (***P < 0.001), *r_G_ =* genetic correlation with 95% highest density posterior interval in parentheses below.

We collected four fecundity and body weight phenotypes from 143 DGRP lines by vial: total number of female progeny, total number of male progeny, and their respective mean weights (in milligrams). We found inter-line differences for all four phenotypes measured (Figure 1A), as well as strong phenotypic and genetic correlations between the phenotypes (Figure 1B). The estimated broad-sense heritability of the vial mean female weight (0.64, 95% HPDI: 0.55–0.72) was lower than for the vial mean male weight (0.73, 95% HPDI: 0.65–0.80). Both weight phenotype heritabilities were higher than previously estimated heritabilities for body weight (Jumbo-Lucioni *et al.* 2010; Nelson *et al.* 2016). The heritabilities of the total number of female progeny (0.47, 95% HPDI: 0.37–0.57) and male progeny (0.48, 95% HPDI: 0.39–0.58) were higher than the heritabilities previously estimated on number of eggs laid (Durham *et al.* 2014) (Supplementary Table S1).

**Figure 2:**
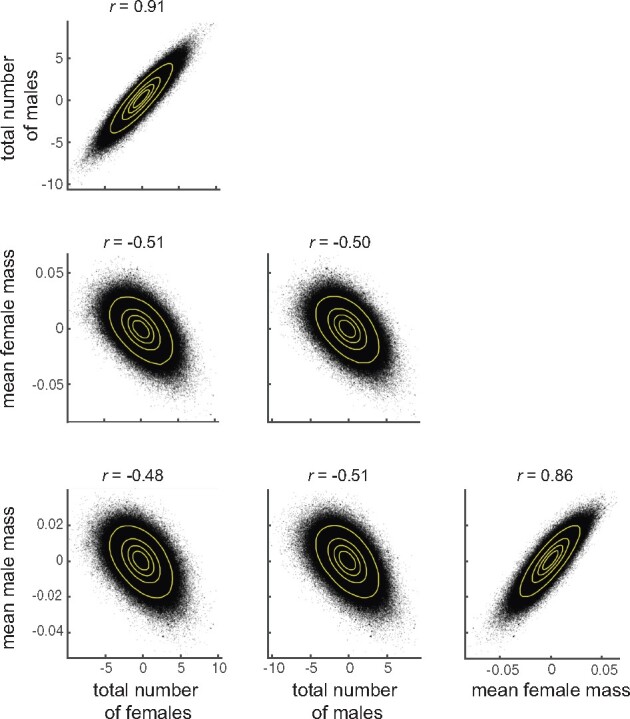
Scatter plots of variant effect sizes for ∼2 million DGRP variants. Each point is a variant plotted at 5% opacity. Lines are contours containing 25%, 50%, 75%, and 95% of the points. Variant effects were calculated as one-half of the difference between means of the major and minor alleles (Huang *et al.* 2014).

We carried out a GWAS on each of the four phenotypes. The top-associated variants are reported in Supplementary Table S2. Surprisingly, no overlapping variants were found among the top hits for offspring mass and number, despite strong correlations between traits (Figure 1B). We hypothesized that the high correlation may arise from many variants of small effect affecting our phenotypes. Such variants would not necessarily be statistically significant variants for both mass and number, but their estimated effects on both phenotypes might be correlated, nevertheless. We estimated the effect size of 1,907,562 DGRP variants on each of the four measured traits and examined the correlation between these effects (Figure 2). We found correlations of variant effects across our phenotypes closely mirroring the genetic and phenotypic correlations [0.48 < abs(*r*) < 0.91]. These relations were supported by many thousands of variants, consistent with the hypothesis that the genetic and phenotypic correlations are due to the small contributions of many variants.

Consistent with a tradeoff imposed by resource limitation, we found that the number of offspring was negatively correlated with the offspring weight (Figure 1B). The strong correlations between the offspring number and weight phenotypes prompted us to combine the four measurements into a single metric. We did this using principal component analysis and termed it the offspring index. Such an approach could provide more power to detect important pleiotropic variants in a GWAS than analyzing each trait independently. The first principal component explained 71% of the variance in the data and is negatively loaded for offspring number and positively loaded for offspring weight (Supplementary Table S3). A negative index value indicated many low-weight offspring, a positive index value indicated few high-weight offspring, and an index value close to zero indicated a balance between offspring number and weight.

We used this offspring index for a GWAS. Thirty variants were associated with the offspring index using a threshold of *P *<* *1E−5 (Table 1). The third chromosome had 12 significant variants, while 16 were located on the second chromosome and only 2 on the X chromosome (*χ*^2^ test, *P *=* *0.32). Most of the variants (23/30) were within 1000 bp of a gene; roughly half of these (14/23) were located in introns. Five of the associated variants were present in genes previously associated with fecundity (Durham *et al.* 2014), which is more overlap than expected given a random set of candidate genes (Supplementary Figure S1A). Among the candidate genes, we did not find significant enrichment for particular biological processes or molecular functions using PANTHER’s Overrepresentation Test with the GO-Slim annotation sets (Mi *et al.* 2019). The QQ plot showed no systematic bias and a slight enrichment (λ_median_ = 1.015) for *P *<* *1E−5 (Supplementary Figure S1B). The linkage disequilibrium heat map revealed no long-distance linkage between variants (Supplementary Figure S1C).

**Table 1:** **Variants significantly associated with the offspring index (** *P* ****<* *1E**−**5)**

Chr	Pos	MAF	Effect	*P* **-value**	Gene	Class
3L	18174169	0.49	0.70	3.4E−07	*hid*	Intron
2L	10356660	0.10	−1.11	7.7E−07	***CG5367***	Upstream (63 bp)
3L	14106797	0.46	−0.60	9.2E−07	***Sox21b***	Del (12 bp—Intron)
3R	5440058	0.10	−1.08	1.0E−06	*CG8312*	Intron
3R	5437737	0.09	−1.10	1.0E−06	*CG8312*	Intron
3R	11212396	0.07	−1.26	1.4E−06	*Rbp*	Intron
3R	10285026	0.05	−1.48	2.0E−06	***cv-c***	Intron
3R	2150285	0.12	−0.98	2.7E−06	*Osi17*	Intron
2R	19814320	0.13	−0.91	3.0E−06	*CG2812*	3ʹ UTR
2L	22137883	0.35	−0.70	3.2E−06	*CG42748*	Intron
2R	8813359	0.29	0.70	3.9E−06	*sug*	Intron
2R	10191983	0.06	−1.33	4.0E−06	*—*	*—*
2R	16280567	0.35	−0.60	4.0E−06	*—*	*—*
2R	10186017	0.13	−0.93	4.2E−06	*Ih*	3ʹ UTR
X	16619471	0.34	0.65	4.3E−06	*CG32572*	Intron
2R	18428402	0.05	−1.39	4.9E−06	***px***	Synonymous
2R	16643265	0.16	−0.82	5.6E−06	*—*	*—*
3L	21865887	0.12	−0.93	5.9E−06	*mub*	Intron
2R	10185377	0.06	−1.33	5.9E−06	*Ih*	Intron
2L	14413190	0.05	−1.32	6.2E−06	*—*	*—*
2L	14413193	0.06	−1.25	6.2E−06	*—*	*—*
3L	16206105	0.16	−0.84	6.4E−06	*CG13073*	Downstream (46 bp)
2R	10354544	0.05	−1.44	6.9E−06	*—*	*—*
3L	16206075	0.15	−0.85	7.1E−06	*CG13073*	Downstream (76 bp)
X	16619495	0.33	0.64	7.4E−06	*CG32572*	Intron
3R	6652348	0.21	−0.68	7.9E−06	*Cad86C*	Upstream (453 bp)
2L	19610094	0.07	−1.14	8.3E−06	***Lar***	Intron
2R	10185828	0.08	−1.10	8.3E−06	*Ih*	Intron
2L	14413263	0.05	−1.36	9.6E−06	*—*	*—*
3R	14116444	0.35	0.65	9.9E−06	*l(3)05822*	Synonymous

Chromosome coordinates represented in dm5 assembly coordinates. MAF = minor allele frequency, Del = deletion, and numbers in parentheses represent the number of basepairs to the closest gene. Genes in bold were previously identified to contain variants associated with age-specific fecundity (Durham *et al.* 2014). Effect of variant on offspring index is unitless, as offspring index is a principal component score, derived from a PCA on scaled features.

### Offspring phenotype differences among lines are stable under different parental densities

**Figure 3:**
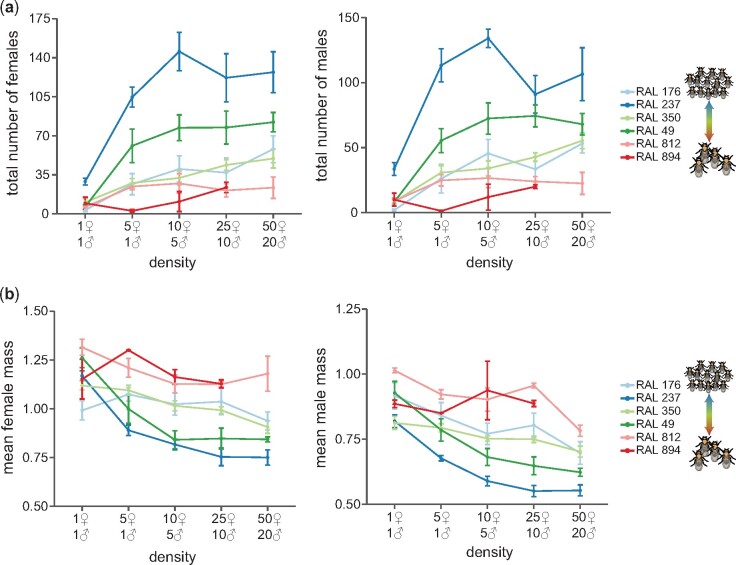
Independent effects of parental density and line on offspring number and weight. (A) Relationship between number of offspring and the density of parents. Each point represents the mean phenotype for a line at a particular density and errors bars show ± 1 SE (*n *=* *2–7). (B) Relationship between mean offspring weight and density of parents.

We measured the variation in offspring index under a specific parental density during egg-laying. To assess the generality of our findings, we examined whether differences among DGRP lines in offspring phenotypes would persist under different parental densities. We chose six lines from our screen that were representative of negative offspring index (many low-weight offspring), intermediate offspring index, and positive offspring index (few high-weight offspring) to assay for offspring phenotypes at different densities of parents during egg-laying (Figure 3). We found that parental density was a significant predictor of offspring number (*χ*^2^ test; females: *P *=* *1.8E−7, males: *P *=* *4.7E−6) and offspring weight (*χ*^2^ test; females: *P *=* *2.2E−12, males: *P *=* *3.1E−12). As expected, increasing parental density increased offspring number and decreased offspring weight, though the effect of increasing parental density increased sublinearly for most lines. After including density as a predictor, we saw that the DGRP line still had a significant impact on offspring number (*χ*^2^ test; females: *P *=* *2.6E−5, males: *P *=* *1.8E−5) and offspring weight (*χ*^2^ test; females: *P *=* *9.5E−8, males: *P *=* *2.7E−7). DGRP lines with positive index (RAL 812: +3.6; RAL 894: +3.8) maintained a low offspring number and high offspring weight under different densities. RAL 237, a DGRP line with a negative index (−2.9), had consistently high offspring numbers and low offspring weights. Surprisingly, RAL 176, a DGRP line with a strongly negative index (−3.2) yielded offspring with weights and counts similar to RAL 49, a line with an index close to zero (−0.04). We did not detect significant line-by-density interactions for any phenotype (*χ*^2^ test; female number: *P *=* *0.66, female weight: *P *=* *0.86, male number: *P *=* *0.54, male weight: *P *=* *0.57).

### Functional validation of associated variants

**Figure 4:**
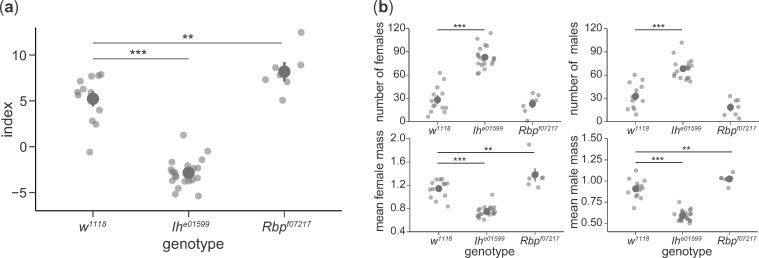
Candidate gene validation using PBac{RB}*Ih^e01599^* (*n* = 10) and PBac{WH}*Rbp^f07217^* (*n* = 7), compared to their genetic background control, *w^1118^*. (A) Offspring index by genotype. (B) Impact of gene disruption on individual phenotypes. In both panels, dark gray points and bars show the mean ± 1 SE; lighter gray points are replicates. Significance levels: *P <* *0.05, **P <* *0.01, ***P <* *0.001. *P*-values were calculated using Dunnett’s test with a family-wise confidence level of 95%.

We chose six candidate genes to validate for involvement with our fecundity phenotype—*hid*, *Sox21b*, *Rbp*, *CG8312*, *mub*, and *Ih*. Genes for validation were chosen based on having a highly associated variant and availability of mutant lines. We used mutant lines from the Exelixis gene disruption panel, which contain *piggyBac* inserts in the genes of interest, to validate our candidate genes (Figure 4). For four of the six candidate genes (*hid*, *Sox21b*, *CG8312*, and *mub*), we found that the available mutations severely impacted pupal and adult viability, to the point where we were unable to generate a stable homozygous line to use in our validation experiments (Table 2). With the remaining genes, *Rbp* and *Ih*, we found significant, opposite effects on the offspring index, with the *Ih* insertion strongly decreasing the offspring index, while the *Rbp* insertion slightly increased it (Figure 4A). Examining component phenotypes (Figure 4B), we saw that the *Ih* insertion significantly increased the number of offspring and decreased mean offspring weight, for offspring of both sexes. Disrupting *Rbp* did not significantly affect the number of offspring, but there was a modest, but statistically significant, increase in offspring weight (13% for males and 21% for females). Both *Ih* and *Rbp* play a role in nervous system function. *Ih* encodes a voltage-gated potassium channel, and *Ih* mutants show defects in locomotion, proboscis extension, circadian rhythm, and lifespan (Chen and Wang 2012; Gonzalo-Gomez *et al.* 2012). *Rbp* encodes a protein involved in the organization of the presynaptic active zone and is instrumental in proper vesicle release (Liu *et al.* 2011)—mutations in *Rbp* can result in neurological and locomotor defects, and in some cases, lethality.

**Table 2:** Homozygous mutant genotypes used in candidate gene validation and their fecundity phenotypes and gene functions

Genotype	Pupae	Adult survival	Fecundity phenotype	Gene function
PBac{PB}*hid^c01591^*	Yes	**No**	*—*	BIR domain-binding protein; apoptosis regulator
PBac{WH}*Sox21b^f06429^*	Yes	**Low**	*—*	Transcription factor; transcription regulation and development
PBac{WH}*Rbp^f07217^*	Yes	Yes	+ offspring weight	RIM-binding protein; presynaptic active zone organization
PBac{RB}*CG8312^e01204^*	Yes	**Low**	*—*	Transcription regulation
PBac{WH}*CG8312^f02825^*	Yes	**No**	*—*	Transcription regulation
PBac{WH}*mub^f02647^*	**No**	*—*	*—*	Regulation of RNA splicing
PBac{WH}*Ih^f01485^*	Yes	**Low**	*—*	Voltage-gated K^+^ channel;
PBac{RB}*Ih^e01599^*	Yes	Yes	+++ offspring number	Voltage-gated K^+^ channel
			− − − offspring weight	

Gene function information was retrieved from FlyBase (Thurmond *et al.* 2019). Number of + or − is a qualitative representation of how much the measured phenotype increased or decreased as compared to the control genetic background line (only statistically significant differences are shown).

For *Ih*, we had multiple mutant alleles whose effects could be compared. *Ih^e01599^* was a viable line with increased offspring count, while *Ih^f01485^* had impaired adult viability (Table 2). While both insertions are in introns, *Ih* transcription has been shown to be disrupted in *Ih^f01485^*, but not in *Ih^e01599^* (Chen and Wang 2012). The effect of the insertion on the final protein function of *Ih^e01599^* is unknown, but RT-PCR shows that intronic insertion in *Ih^f01485^* results in a null mutation (Hu *et al.* 2015).

### Comparison with other DGRP life-history studies

We examined correlations between phenotypes measured in this study and those measured in other DGRP studies that might pertain to fecundity. We looked at the following phenotypes: starvation resistance (Mackay *et al.* 2012), chill coma recovery time (Mackay *et al.* 2012), food intake (Garlapow *et al.* 2015), fecundity and body size (Durham *et al.* 2014), nutritional indices and weight (Unckless *et al.* 2015), and developmental time and egg-to-adult viability under different densities (Horváth and Kalinka 2016). Using a false discovery rate correction with all possible comparisons, we found that offspring weight measured in our study was significantly correlated with body size and mean weight measurements made in previous DGRP studies (Table 3). There was no correlation found between our measurements of total progeny number and fecundity measurements (*P *>* *0.1 for all comparisons). In addition, there was no correlation between progeny weight and food intake (*P *>* *0.1 for all comparisons). Though we found positive correlations between female starvation resistance and female (*r = *0.18, *P = *0.028) and male (*r = *0.17, *P = *0.046) weight measured in our study, these correlations were no longer significant after correcting for multiple tests. In addition, we found a negative correlation between number of offspring and male and female development time under a high larval density treatment [measured previously in 31 DGRP lines (Horváth and Kalinka 2016)], but positive correlation between offspring weight and development time. Egg-to-adult viability under high larval density treatment was positively correlated with offspring number and negatively correlated with offspring weight. Though we observed a trend in the relationships between development time, viability, and our phenotypes, only a few correlations were nominally significant, and none remained significant after the multiple testing correction.

**Table 3:** Correlations of phenotypes measured in our study (measured) with traits measured in previous DGRP studies (comparison)

Measured	Comparison	*r*	*q*-value	Reference
Mean weight (♂)	Body size (−y)	0.26	4.6E−2	Durham *et al.* (2014)
Mean weight (♂)	Body size (+y)	0.28	2.9E−2	Durham *et al.* (2014)
Mean weight (♀)	Mean weight (♂−)	0.41	3.5E−4	Unckless *et al.* (2015)
Mean weight (♂)	Mean weight (♂−)	0.39	6.9E−4	Unckless *et al.* (2015)
Total number (♂)	Mean weight (♂+)	−0.30	2.9E−2	Unckless *et al.* (2015)
Mean weight (♀)	Mean weight (♂+)	0.44	1.4E−4	Unckless *et al.* (2015)
Mean weight (♂)	Mean weight (♂+)	0.41	3.5E−4	Unckless *et al.* (2015)
Mean weight (♀)	Mean weight (♂^cb^)	0.46	6.4E−5	Unckless *et al.* (2015)
Mean weight (♂)	Mean weight (♂^cb^)	0.43	1.4E−4	Unckless *et al.* (2015)

Only correlations that remained significant after multiple testing correction are presented. Symbols are as follows: “−y” denotes a low yeast diet, “+y” denotes a high yeast diet, “+” denotes a high glucose diet, “−” denotes a low glucose diet, and “cb” = overall effect when the data from the glucose diets are combined. *P*-values were transformed into *q*-values using the Benjamini−Hochberg method to correct for multiple tests across all comparisons.

## Discussion

We investigated whether there was a genetic basis for the tradeoff between adult offspring number and weight under space and resource limitation. We found that DGRP lines varied in the number of offspring produced and their mean weight, with numbers negatively genetically correlated with weight, likely due to small pleiotropic effects of many variants. Using a combined “offspring index” derived from the first principal component of our offspring phenotypes, we identified variants associated with variation in offspring index (*i.e.*, variation in the tradeoff between many low-weight offspring to few high-weight offspring). We examined the effects of mutation on six candidate genes and found that for all tested insertion alleles of *hid*, *Sox21b*, *CG8312*, and *mub*, as well as one allele of *Ih*, gene disruption caused phenotypes ranging from pupal lethality to low adult survival. Disruption of *Rbp* caused a small increase in offspring weight and an insertion allele of *Ih* caused a large increase in offspring number coupled with a decrease in offspring weight. When comparing our measured phenotypes to life-history phenotypes measured in other DGRP studies, we found consistency in our body weight measurements and other measurements of weight and body size, but surprisingly, we did not find a relationship between offspring number and previous measures of fecundity.

**Figure 5:**
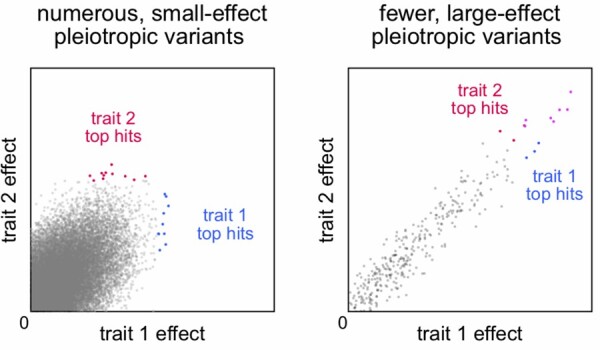
Overlap between top variants associated with two different phenotypes is not guaranteed with highly polygenic genetic architecture. (Left) Points in the scatter plot depict the effect sizes of numerous moderately pleiotropic effects, while the colored points represent the (nonoverlapping) top 10 variants associated with variation in example traits 1 and 2, respectively. (Right) As previous, but for a genetic architecture of fewer pleiotropic variants of larger effect. The top variants overlap (magenta) in this scenario.

We found that, similar to fecundity as measured by number of eggs laid (Durham *et al.* 2014), there is a polygenic basis to offspring number and weight. While several of the candidate genes we found were previously associated with fecundity (Durham *et al.* 2014), most were not. When we examined the top variants associated with each individual trait, we did not find any overlapping variants between offspring mass and number, despite the high genetic and phenotypic correlations between these measures. When we examined the correlation of estimated variant effects, we found a highly significant, modest correlation among many thousands of variants. Because so many variants are associated with both traits, and because the correlation of their effects is not extremely strong, the lists of top variants for each trait are not expected to be overlapping (Figure 5). Overlap would be expected if there were a few contributing variants of large effect or the correlation of effects among many variants were extremely tight. The latter is more the case with the correlation of variant effects between female and male offspring mass and number, where we do observe a few overlapping variants among the top hits (Supplementary Table S2). Among our candidate genes, we were not able to find significant enrichment of genes involved in particular biological processes or molecular functions, though given the limited number of genes used in this analysis, only a very strong enrichment could have been significant. While only two of the six of the candidate genes tested in our validation experiments are annotated as having roles in developmental processes, we found that for most genes tested, mutation disrupted the survival of either the larval, pupal or adult stages severely enough to prevent us from even measuring offspring number and weight.

The omnigenic model of genetic architecture (Boyle *et al.* 2017; Zhang et al. 2019) could explain the functionally varied suite of genes associated with offspring number and weight phenotypes. One of the predictions of the omnigenic model is that essentially all genes contribute to a trait through their expression at some appropriate developmental point or in a particular tissue. This leads to many loci in diverse genes being weakly associated with the trait of interest. The omnigenic model also predicts that there should be a network of genes whose action is essential and that have correspondingly higher effect sizes. Interestingly, even though we observed that disruptions to our candidate genes led to strong effects, we did not find an enrichment for correlated expression among them (see Supplementary File S1), *i.e.*, they did not appear to be part of known transcriptional networks. We were also unable to find enrichment for particular biological pathways or molecular processes among our candidate genes. Since we were unable to identify a network uniting our candidate genes, our results are not strictly consistent with an omnigenic model, but perhaps reflect what was proposed in Fisher’s “infinitesimal model” (Turelli 2017), in which a quantitative trait is made up of tiny contributions of essentially all genes. We observed strong correlations of variant effects between measured phenotypes across almost 2 million DGRP variants (Figure 2), lending further support to the “infinitesimal” model.

We found that insertion mutations in *Rbp* and *Ih* caused opposite phenotypes. Mutations in *Ih* increased the offspring number and decreased offspring weight, presumably due to the constraints of the vial. *Ih* had not been previously implicated in fecundity phenotypes as measured by egg-laying. While that does not preclude that *Ih* mutants may lay more eggs, a higher egg-to-adult viability for *Ih* mutants could also lead to the increase in offspring number. Disruption of *Rbp* only increased weight without significantly affecting offspring number. A small effect on offspring number could have been obscured by limited sample size, but our results still indicate that the weight increase was more prominent than the decrease in offspring number. *Rbp* is involved in neurological function and locomotion. This role could affect body weight through a number of mechanisms including foraging, feeding, gut peristalsis, etc. A decoupling of weight from offspring number shows that there is an independent axis where offspring weight can increase even though the level of larval competition and other density effects remain the same. This notion is supported by the second principal component of our dataset, which is positively loaded for both weight and offspring number, indicating that a tradeoff between the two is not inevitable (Supplementary Table S3).

We observed that even within the same gene, different disruptions can lead to different phenotypes. Both the *Ih^e01599^* and *Ih^f01485^* alleles are intronic insertions that affect most transcripts (Chen and Wang 2012; Hu *et al.* 2015), but *Ih^f01485^* shows impaired viability, while *Ih^e01599^* shows an increase in offspring number. The *Ih^f01485^* allele was reported to eliminate expression of all *Ih* transcripts (Hu *et al.* 2015). In contrast, the *Ih^e01599^* allele was reported as having wild-type levels of expression (Chen and Wang 2012). Based on its insertion position, the *Ih^e01599^* allele would affect 8 of the 11 *Ih* transcripts. The phenotype difference we observed between these alleles could depend on specific transcripts. These results highlight a general caveat about using mutant lines to make conclusions about the exact role of the gene in determining the phenotype, rather than a more general conclusion about whether or not the gene plays a role at all.

When we collected offspring from parents at different densities, we found a sublinear increase in the number of progeny as the density of parents increased, consistent with the effects of resource limitation. We also found that offspring phenotypes were strongly determined by DGRP line at all densities. To first approximation, DGRP lines that produce fewer larger offspring continued to produce fewer larger offspring regardless of the number of parents allowed to oviposit in the vial. There appear to be parallels between the variation in offspring phenotypes among DGRP lines and *r* and *K-*selection (MacArthur and Wilson 2001). In an ecological context, an *r-*selected species is one that produces many offspring with low chances of survival. A *K-*selected species invests in few high-quality offspring in order to be able to compete in more crowded environments with limited resources. If the *r*/*K* framework plays out as a plastic trait within a genotype, we might expect lines to converge on smaller offspring in conditions of higher crowding. We observed no such convergence, nor indeed any significant line-by-density effects. The genetically determined variation in offspring phenotypes among our lines seems to reflect a within-species continuum between *r* and *K-*selected types, perhaps consistent with a Pareto frontier of equivalently fit phenotypic combinations. Given that we are studying this trait in a collection of inbred lines meant to capture the genetic variation of an outbred population, it is also possible that outbred *D. melanogaster* populations exhibit less variation on this continuum, while the inbred lines lock in diverse phenotypes based on their varied genetic compositions.

Comparing our phenotypes to those measured in other DGRP studies, we found correspondence between our weight measurements and body weight/size measurements from other studies, affirming that DGRP line phenotypes can remain consistent across different study environments. We note that our approach, by design, only controls for parental density and not larval density, which would reduce the concordance between the findings of our study and other DGRP studies. As such, we find that the heritability of body weight measured in this study is higher than that measured previously (Jumbo-Lucioni *et al.* 2010), where larval density was controlled. Offspring body weight can be considered as the integrated result of several processes, including egg fertilization and hatching. Maternal genetic effects, via egg size, play a role in positively regulating viability and hatchling weight, though the direct effect of egg size on offspring weight was not clearly established (Azevedo *et al.* 1997). In our study, we did not control for variation in these other intermediate phenotypes among the DGRP genotypes, as we were interested in their final integrated effect on body weight. Our observation of higher heritability of body weight, compared to other studies, could reflect several factors. Gene-by-environment interactions or genetic drift in the mapping populations could affect the measures of heritability, or the final body weight phenotype in our study could reflect an effective average of the contributions of intermediate phenotypes, like egg fertilization and egg hatching. We speculate that this could result in a higher signal-to-noise ratio in the relationship between genetic variation and progeny weight variation, compared to the signal-to-noise of intermediate traits. It is also important to note that we did not control for the masses of the female parents, a possible maternal effect, when examining variation in our focal phenotypes. We used groups of 10 females reared in density-controlled bottles to generate the offspring scored for the GWAS, which would average out differences within lines, but not between lines. There may also be among-genotype variation in parental female mass that is nongenetic, *e.g.*, due to parental rearing conditions. Nongenetic variation would add noise to our measurements, though we still observed robust genetic correlations in our study.

To our surprise, we did not see a correlation between our fecundity measure (the number of offspring) and prior fecundity measures (number of eggs laid). This may be indicative of substantial line-to-line variation in mortality post-egg-laying but pre-eclosion. Alternatively, there could be a positive correlation between egg survival (fertilization and hatching) and offspring number, but that a negative correlation between number of eggs laid and egg survival. The combination of these two mechanisms could yield no correlation between number of eggs and offspring number. Another possible explanation for the lack of correlation could be fecundity having been previously assayed with individual females (Durham *et al.* 2014), whereas we housed females in groups of 10 for our assay. The number of eggs laid per female was shown to decrease in more crowded conditions (Barker 1973; Ohnisni 1976), along with differential genotype effects (Ohnisni 1976), could lead to the lack of correlation observed. We also observed that our measure of fecundity (producing more offspring) was correlated with a lower developmental time and higher egg-to-adult viability under a high-density treatment in previous studies. Though these correlations were not significant after a multiple testing correction, it does indicate that lines producing more offspring may have adapted to high-density lab rearing conditions (Santos *et al.* 1994; Horváth and Kalinka 2016).

Overall, our results point to a polygenic basis for offspring number and weight. Validation of six candidate genes implicates diverse biological processes in controlling adult stage viability. Combining our results with results from studies on other *Drosophila* life-history traits, we find support for the idea that traits closely related to fitness (*e.g.*, offspring number) may be influenced by a large set of genes, perhaps ultimately encompassing the vast majority of functional genes.
